# Dynamic Profiling: Modeling the Dynamics of Inflammation and Predicting Outcomes in Traumatic Brain Injury Patients

**DOI:** 10.3389/fphar.2016.00383

**Published:** 2016-11-01

**Authors:** Gregory Constantine, Marius Buliga, Qi Mi, Florica Constantine, Andrew Abboud, Ruben Zamora, Ava Puccio, David Okonkwo, Yoram Vodovotz

**Affiliations:** ^1^Department of Mathematics and Department of Statistics, University of PittsburghPittsburgh, PA, USA; ^2^Center for Inflammation and Regenerative Modeling, McGowan Institute for Regenerative Medicine, University of PittsburghPittsburgh, PA, USA; ^3^Department of Mathematics, University of PittsburghBradford, PA, USA; ^4^Department of Sports Medicine and Nutrition, University of PittsburghPittsburgh, PA, USA; ^5^Department of Applied Mathematics and Statistics, Johns Hopkins UniversityBaltimore, MD, USA; ^6^Department of Surgery, University of PittsburghPittsburgh, PA, USA; ^7^Department of Neurological Surgery, University of PittsburghPittsburgh, PA USA

**Keywords:** TBI, TBI outcome, Inflammation, mathematical modeling, precision medicine

## Abstract

Inflammation induced by traumatic brain injury (TBI) is complex, individual-specific, and associated with morbidity and mortality. We sought to develop dynamic, data-driven, predictive computational models of TBI-induced inflammation based on cerebrospinal fluid (CSF) biomarkers. Thirteen inflammatory mediators were determined in serial CSF samples from 27 severe TBI patients. The Glasgow Coma Scale (GCS) score quantifies the initial severity of the neurological status of the patient on a numerical scale from 3 to 15. The 6-month Glasgow Outcome Scale (GOS) score, the outcome variable, was taken as the variable to express and predict as a function of the other input variables. Data on each subject consisting of ten clinical (one-dimensional) variables, such as age, gender, and presence of infection, along with inflammatory biomarker time series were used to generate both multinomial logistic as well as probit models that predict low (poor outcome) or high (favorable outcome) levels of the GOS score. To determine if CSF inflammation biomarkers could predict TBI outcome, a logistic model for low (≤3; poor neurological outcome) or high levels (≥4; favorable neurological outcome) of the GOS score involving a full effect of the pro-inflammatory cytokine tumor necrosis factor-α and both linear and quadratic effects of the anti-inflammatory cytokine interleukin-10 was obtained. To better stratify patients as their pathology progresses over time, a technique called “Dynamic Profiling” was developed in which patients were clustered, using the spectral Laplacian and Hartigan’s k-means method, into disjoint groups at different stages. Initial clustering was based on GCS score; subsequent clustering was performed based on clinical and demographic information and then further, sequential clustering based on the levels of individual inflammatory mediators over time. These clusters assess the risk of mortality of a new patient after each inflammatory mediator reading, based on the existing information in the previous data in the cluster to which the new patient belongs at the time, in essence acting as a “virtual clinician.” Using the Dynamic Profiling method, we show examples that suggest that severe TBI patient neurological outcomes could be predicted as a function of time post-TBI using CSF inflammatory mediators.

## Introduction

In the United States alone, traumatic brain injury (TBI) accounts for an estimated 3.5 million emergency department visits, hospitalizations, and death (Coronado et al., 2012). Despite research leading to innovative treatments and standardized care, TBI-related morbidity remains a major cause of disability in the United States with an estimated 5.3 million people living with long-term cognitive and psychological impairments each year (Selassie et al., 2008). Although improvements in post-TBI mortality have been seen in recent years (Spiotta et al., 2010; Coronado et al., 2012), morbidity following a severe TBI remains extremely high, frequently accompanied by long-term disability (Selassie et al., 2008), and costly, with an estimated economic cost at $76.5 billion, including direct medical costs and indirect costs (e.g., lost wages, and lost productivity and non-medical expenditures) (CDC, 2013).

The primary injury of a severe TBI is heterogeneous and may result in morphological damage to cerebral structures due to physical trauma and may include bleeding within the intracranial cavity, diffuse axonal injury and brain tissue swelling (DeKosky et al., 2013). The response to the initial TBI leads to neurochemical changes that have direct pathogenic effects on regional cerebral blood flow (CBF), blood-brain barrier function, cerebral metabolism, ion homeostasis, and/or brain tissue that may be further impacted by secondary injuries, such as hypotension and hypoxia. This secondary damage develops over time, beginning immediately after injury and evolving over hours, days, and weeks after the initial trauma. These cellular and molecular changes initiate both neuroprotective and neurotoxic cascades and ultimately impact recovery (Selassie et al., 2008). In particular, inflammatory responses that occur post-TBI may greatly impact neurological outcome. In autopsy tissues from patients that succumbed to severe TBI, acute microglial and macrophage activity was evident 2–10 days after injury (Velazquez et al., 2015). Increased levels of inflammatory proteins in CSF and serum indicative of an inflammatory response have been noted within hours to days following an injury (Newell et al., 2015; Shan et al., 2016). Although acute inflammation after TBI may be neuroprotective to assist in repair and recovery (Corps et al., 2015; Michell-Robinson et al., 2015; Chen and Trapp, 2016); there is additional evidence that an exaggerated or lack of clearance of inflammatory activity which may result for an extended period after injury is associated with deleterious effects and poorer clinical outcomes (Schwarzmaier and Plesnila, 2014; Corps et al., 2015; Hinson et al., 2015; Lozano et al., 2015; Witcher et al., 2015; Karve et al., 2016).

Current predictors of outcome after severe TBI are neither sufficiently sensitive or specific to be used for clinical decision making in the acute recovery period (Gao and Zheng, 2015). Improving individual outcome after severe TBI will require interactive measures of acute injury (cellular level) and response to injury. We believe the balance of pro- and anti-inflammatory mediators influences survival after severe TBI. We propose that this balance, or alterations in this balance, can be used to develop predictive models of cellular level damage and survival after TBI. Because both the response to TBI and the inflammatory response are complex processes, more refined analytic techniques are needed to predict outcomes and identify the role of inflammatory processes after TBI in a clinical setting. Temporal and patient-specific variations in the response profile, multi-mechanistic injury and the interaction of molecules in variable tissue level environmental states further this complexity. Such complexity and individual variability in response require an analytic procedure that can account for temporal changes in response, identify patterns in the resultant data, and determine dynamic patterns that accurately predict recovery.

In the present study, we produced data-driven statistical models for TBI, based on serial measurements of inflammatory mediators in the cerebrospinal fluid (CSF) of severe TBI patients. Our goal was to develop models that relate the initial severity of injury, along with the patient demographic data and clinical and inflammatory mediator biomarker data, to their overall, dynamic state of health. This paper first describes a statistical analysis of the data, followed by a clustering module that accomplishes subject profiling over time based on incrementally accumulating inflammation of biomarker data. The power of the method is then evaluated. We develop also a logistic model based, in part, on the profiling method to predict the patient state of health from the available clinical and inflammatory mediator data collected during the acute period of hospitalization. We suggest that this *Dynamic Profiling* approach may be used as a diagnostic tool during the acute care period to provide additional predictive power for rehabilitative trajectory recovery.

## Materials and Methods

### Traumatic Brain Injury Patients

Severe TBI patients were enrolled prospectively in this University of Pittsburgh Institutional Review Board-approved study upon meeting inclusion criteria judged by the on-call neurosurgeon. Informed consent was obtained by the legal authorized representative prior to study procedures. CSF and blood samples were obtained by trained study personnel for the initial through 5 days of ICU admission. A trained neuropsychological technician obtained the 6- and 12-month Glasgow Outcome Scale (GOS) scores. The patient cohort consisted of 27 TBI patients [20 survivors (18 males/2 females) and 7 non-survivors (6 males/1 female)]. Non-survivors were determined by having a Glasgow Outcome Scale (GOS) score of 1 by 12 month follow up, and had a Glasgow Coma Scale (GCS) score (an estimate of TBI injury severity) of 5.6 ± 0.57 on hospital arrival. Survivors had a similar admission GCS of 6.0 ± 0.24 (**Table 1**), with a GOS score of 2–5.

**Table 1:** General demographics and injury characteristics of TBI patient cohort.

	Survivors	Non-Survivors
Age	34.0 ± 3.1	37.9 ± 5.7
Sex Ratio (M:F)	18:2	6:1
GCS	6.0 ± 0.24	5.6 ± 0.57

### Description of the Data

The data on each subject consisted of two distinct components, namely clinical/demographic data and CSF inflammatory mediator data. Clinical/demographic (one-dimensional) variables included: age, gender, presence of infection, bleeding, surgical decompression, presence of subarachnoid hemorrhage, and initial GCS score. Inflammatory mediator data consisted of acute CSF time series in each of 13 inflammatory mediators. The GCS score quantified the initial brain injury severity on a numerical scale from 3 to 15. The inflammatory mediator time series variables varied in both in length and in the time sequence at which they were collected.

The GOS score was utilized as the outcome variable and was viewed as the response variable to study and predict neurological outcome, as a function of the other input variables. The GOS score quantifies the neurological outcome at 6 and 12 months post-TBI. GOS scores ranged from 1 to 5, with 1 indicating death and higher values indicating a progressively better neurological state of health.

In addition to the clinical and demographic data, data included inflammatory mediator readings on 34 patients. The data are given as time series for the following 13 cytokines/chemokines (assayed using Luminex^TM^ multiplexing technology): IL-1α, IL-1β, IL-2, IL-4, IL-5, IL-6, IL-8, IL-10, IL-13, macrophage inflammatory protein (MIP)-1α, MIP-1β, tumor necrosis factor (TNF)-α, and vascular endothelial growth factor (VEGF). Correlation analysis on the 13 time series found dependencies among the variables.

### Logistic Regression Models

We initially sought to validate our study population against prior studies of circulating inflammation biomarkers (Forde et al., 2014), and to determine if we could utilize CSF inflammatory biomarkers to segregate patient neurological outcomes. Accordingly, we extracted from each time series, associated to a given inflammatory mediator, orthogonal (Chebyshev) polynomial trends up to a specific degree d. The degree d is constant across both inflammatory mediators and subjects. We then utilized these polynomial trends, quantified as one-dimensional variables, as predictors for the GOS score, representing neurological outcome. The degree d was constant across both inflammatory mediators and subjects. Choice of d, typically *d* = 1, 2, or 3, restricts the patients that can be included in the analysis; specifically, we can only include those that have at least d+1 rounds of biomarker readings. Multinomial logistic analytics were explored, as well as probit models. The models selected emerged upon fitting to data, and selecting the statistically significant clinical predictors as well as the orthogonal polynomial time trends of inflammatory mediators. Upon extracting polynomial trends, we performed analyses of the residuals.

When extracting polynomial trends, two options were considered: smoothing the time series and then taking the trends, or taking the trends directly on the unsmoothed data. Though it is common to smooth the data, in this case we observed large differences in the time series response of certain mediators. It seemed reasonable to attempt to capture these changes with polynomials of higher degree, thus allowing extraction of higher order polynomial trends from the unsmoothed data as an option in the analysis. The model was obtained by using 80% of the available data and was tested on the remaining 20%. Ultimately, a logistic model was found as an optimal predictive tool.

### Dynamic Profiling

**FIGURE 1:**
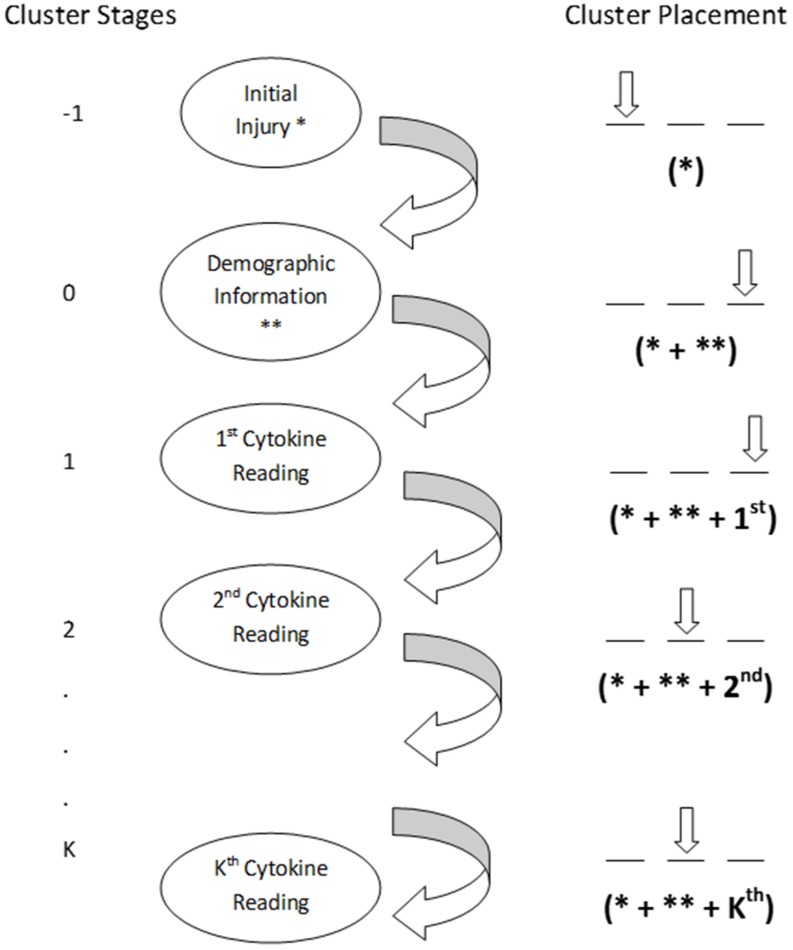
**Clusters are determined by existing patient data, and a particular GOS outcome is associated with each cluster.** As a new patient arrives, they are placed in a cluster at each stage based on what the cluster is examining (found in parenthesis under the clusters). After each round of inflammatory mediator readings, the potential GOS outcome for the patient is predicted by the cluster the patient is in. This figure was previously published in modified form in Vodovotz et al. (2010).

We developed the Dynamic Profiling method as a means of assessing the dynamic course of a TBI patient within the acute care hospital setting (**Figure 1**). In the present application of Dynamic Profiling, a cluster is a subset of severe TBI patients that share similar characteristics. The set of clusters, recalculated after each set of inflammatory mediator readings, forms a partition of the TBI patients. To a given cluster, we associate three statistics based on the GOS score: the number of GOS scores equal to 1 in the cluster (this is the number of patients that died, to which we refer as “red flags”), the average GOS score of the subjects in the cluster, and the standard deviation of the GOS scores in that cluster. The vector of these three statistics is called the “weight” of the cluster. A cluster has a favorable weight if it has a small number of deaths, a high GOS average and a low GOS standard deviation. A useful statistic for the cluster is the probability of death of a patient belonging to that cluster (a “red flag”); it is derived as the ratio of “red flags” to the total number of subjects in the cluster. During the hospital stay the aim is to diagnose and reduce the probability of death, as we pass from stage i clusters to stage i+1.

Clustering, at any stage, was based on Hartigan’s k-means routine (as implemented in R and Splus). Initially (Stage -1), before any inflammatory mediator data are collected on the subject, the algorithm classifies solely in accordance to the initial GCS score. We use as many clusters as seems appropriate for the size of the available data; in the present study, we chose to use three clusters at any stage of clustering. We then introduce the clinical (and demographic) information and produce (three) new clusters; we call these Stage 0 clusters. Across the time series of the inflammatory mediator data we revise the existing clusters, and produce stage i clusters after the i^th^ round of inflammatory mediator readings. The i^th^ round of inflammatory mediator readings refers to the time interval since initial injury during which inflammatory mediator data readings were collected. Variables used to obtain the clusters are the following: initial GCS score, the subset of statistically significant demographic and clinical variables, the statistically significant polynomial trends in the time series of inflammatory mediator readings up to stage i-1 clustering (inclusive), and the inflammatory mediator readings during the current time interval. We note that the number of variables used to cluster on does not increase as we move to higher stage clustering. Indeed, we only use polynomial trends of degree at most d, irrespective of the length of the time series, or, equivalently, irrespective of the stage of clustering. This yields robustness to the clustering process while simultaneously bounding the dimension in which clustering takes place. The clusters weights offer the opportunity of identifying patterns in the inflammatory mediators that yield favorable GOS scores. As a new patient is received we classify him by the severity of initial injury (GCS score) - Stage -1 clustering. We classify next by adding the demographic and clinical variables - Stage 0 clustering. In the i^th^ time interval after the initial injury, we classify in accordance to previous inflammatory mediator trends and current inflammatory mediator readings; this is Stage i clustering. At each Stage, the fraction of “red flags” (deaths) in the cluster in which the new patient falls, estimates the probability of death of the patient. The procedure lends itself easily to a Bayesian approach by placing a prior distribution (of probability of death) on existing clusters based on known medical expertise not pertaining to the data at hand. This is then updated by the observed data through the Dynamic Profiling method described above. The resulting posterior distribution encapsulates both the medical expertise as well as the observed probabilities of death within the data. We thus obtain parametric models from both the Chebyshev orthogonal polynomial fits to the time series data, as well as the Bayesian prior and posterior distributions. This algorithm is implemented as a module in the R language.

## Results

### Logistic Regression Modeling of Post-TBI Inflammation Data

We initially sought to determine if CSF inflammatory mediators are associated with neurological outcomes post-TBI. Our initial approach to testing this hypothesis involved extracting from each time series orthogonal polynomial trends up to a specific degree d, associated to a given inflammatory mediator. The degree d was constant across both inflammatory mediators and subjects. We then used these polynomial trends, quantified as one-dimensional variables, as predictors for the GOS score. Thus, we combined the clinical variables with the time series data to produce a total of 10 clinical and 13(d+1) polynomial trends as potential predictors for the GOS score. The 10 + 13(d+1) one-dimensional variables were used to produce a predictive model for GOS score. Extraction of trends as orthogonal Chebyshev polynomials has the advantage of relating time series of differing lengths. Specifically, the existence of a linear or quadratic trend in a time series of length 5 and another of length 8 provides a meaningful comparison of the two series in spite of their differing lengths.

We then explored multinomial logistic as well as probit models. The models emerged upon fitting to data, and subsequent selection of the statistically significant clinical predictors as well as the orthogonal polynomial time trends of inflammatory mediators. Upon extracting polynomial trends, we carried out a study of the residuals. An issue of concern is the large variations observed in the residuals of certain inflammatory mediator readings at certain time intervals. We have no explanation for this, other than possible significant clinical interventions, such as administration of drugs or surgical procedures that are not recorded in the data.

The model was obtained by using 80% of the available data and was tested on the remaining 20%. Ultimately, a logistic model was found as an optimal predictive tool. The model involves only two inflammatory mediators, TNF-α (a canonical pro-inflammatory mediator) and IL-10 (a canonical anti-inflammatory mediator). Specifically, the log-odds ratio is expressed as an additive model containing the full TNF-α effect in addition to a linear and quadratic effect in IL-10.

The significance of the logistic model, along with its coefficients, is summarized in **Table 2**. Furthermore, the fitted probabilities of the logistic model are given in **Table 3**. The first column records the patient number, the second is the binary GOS score outcome (1 indicates survival, and 0 indicates death), while the third column gives the predicted probability of survival based on the model specified above. In the present study, we were only able to use *d* = 2 or 3 in the predictive models we developed, due to the difficulty in obtaining a larger number of CSF measurements in TBI patients, precluding the construction of a statistical model with a large number of parameters that a higher value of d would entail.

**Table 2:** Parameter estimates of the logistic model predictive of GOS score.

	value	Standard Error	*t*-value
(Intercept)	-9.29	4.77	-1.95
TNFa.L	1.68	0.79	2.14
TNFa.Q	0.54	0.27	1.98
TNFa.C	-1.04	0.49	-2.14
IL10.L	-0.09	0.11	-0.82
IL10.Q	0.19	0.12	-1.57

**Table 3:** Observed and fitted probabilities of survival using the logistic model.

Patient number	1	2	3	4	5	6	7
GOS outcome	1	0	1	0	1	1	1
Probability of survival	1.00	0.87	0.55	0.29	0.78	0.95	0.89
Patient number	8	9	10	11	12	13	14
GOS outcome	0	1	1	1	1	1	0
Probability of survival	0.12	0.97	1.00	0.97	0.90	0.24	0.00
Patient number	15	16	17	19	21	22	28
GOS outcome	1	1	1	1	1	1	0
Probability of survival	0.90	0.58	0.99	0.75	0.99	0.99	0.62
Patient number	29	30	31	33	34	35	
GOS outcome	0	1	1	1	1	0	
Probability of survival	0.61	1.00	0.88	1.00	0.77	0.38	

### Dynamic Profiling of Patient-Specific Outcomes Post-TBI

Logistic regression modeling can account for the properties of sub-groups of TBI patients (such as survivors vs. non-survivors, or low score GOS vs. high). However, a key piece of information missing relates to the *probability over time* of the occurrence of such outcomes. We hypothesized that changes in this dynamic probability of survival vs. non-survival are related to the dynamics of the inflammatory response as well as to factors intrinsic to the patient (i.e., key demographic indicators) and to the injury (i.e., metrics such as GCS score). We therefore sought to develop a *dynamic* tool for assessment of TBI based on demographic and inflammation biomarker reading time series taken during stay in the emergency room and in the hospital. To address this goal, we developed the Dynamic Profiling method. This method allows estimation of certain probabilities of recovery for a patient at any time during the hospital stay based on the knowledge gained from the previously modeled data at all prior sampling times. Specifically, from the available data we developed profiles by clustering subjects into disjoint groups and seeking predictive techniques that exploit both the similarities within clusters as well as differences among clusters (**Figure 1**).

We note that the GOS score is the statistic that assesses outcome and that the GCS score assesses initial injury. The Pearson correlation between these two scores is only 0.06 across subjects. The correlation becomes 0.56 across surviving subjects, however (not shown; Abboud et al., 2016). Accordingly, we counted the deaths within a cluster (or equivalently, upon dividing to the cluster size, the probability of death within a cluster) as the primary component of the cluster weight. There are at least 13 GCS score values possible, but we use as many clusters as seems appropriate for the size of the available data. In the present study, we chose to use three clusters at any stage of clustering.

**FIGURE 2:**
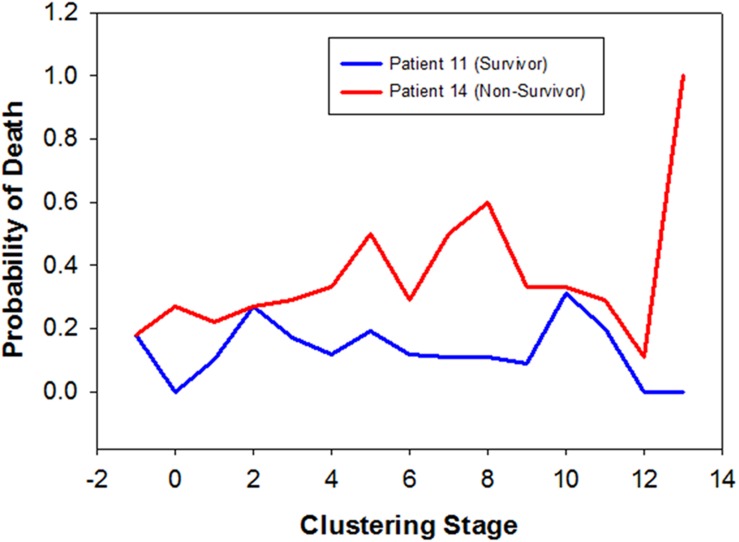
**Dynamic Profiling of TBI survivor vs. non-survivor.** The three clusters are determined by existing patient data, and a particular GOS outcome is associated with each cluster. As a new patient arrives, he is placed in a cluster at each stage based on what the cluster is examining (found in parenthesis under the clusters). The potential GOS outcome for the patient can be predicted by which cluster the patient is in. Red curve indicates the probability of death for Patient 14 (non-survivor). Blue curve indicates the probability of death for Patient 11 (survivor). Data is available in **Table 4**. This figure was previously published in modified form in Vodovotz et al. (2010).

To illustrate the process of dynamic profiling, we highlight subjects 11 and 14 and assess their evolution through the dynamic profiling clusters. The results are summarized in **Table 4** with a graphical display given in **Figure 2**. The subject profiling technique reveals the following information in this case. At Stage -1 clustering (clustering solely on the initial injury GCS score), we see from **Table 4** that subjects 11 and 14 belong to the same cluster (cluster “2”). This is not surprising, since subject 11 has GCS score 6, while subject 14 has GCS score 7 (quite close to each other in ranking).

**Table 4:** Summary of the performance of Dynamic Profiling for two exemplar patients.

Cluster Stage	–1	0	1	2	3	4	5	6	7	8	9	10	11	12	13
Patient 11	0.18	0.00	0.10	0.27	0.17	0.12	0.19	0.12	0.11	0.11	0.091	0.31	0.20	0.00	0
Patient 14	0.18	0.36	0.22	0.27	0.29	0.33	0.50	0.29	0.50	0.60	0.333	0.33	0.29	0.11	1

At Stage 0 clustering, when only clinical and demographic variables (but not data on inflammatory mediators) are used to cluster (in addition to the GCS), we notice that subject 11 belongs to the “best” cluster (which has no deaths). In contrast, subject 14 is in the “worst” cluster (cluster 1, which has chance of death more than a third – 4/11). Demographic data are more favorable to patient 11 who is 29 years old, as contrasted with subject 14 who is 60. In this instance, we know that patient 11 recovers with a GOS score of 4, while patient 14 dies.

Stage 1 clustering starts on the first instance of inflammatory mediator readings. Patients 11 and 14 had 12 rounds of inflammatory mediator readings. As can be read in **Table 4**, and seen graphically in **Figure 2**, we have 12 clusters based on these inflammatory mediator readings, with probabilities of death estimated from the cluster weights. It is worth noting that patient 14, who dies, has uniformly higher chance of death, across all inflammatory mediator readings.

In order to assess the predictive power of Dynamic Profiling, we adopted the following strategy. From the existing subjects, we omitted one subject. The subject omitted is treated as an incoming TBI patient, the treatment evolution of whom will be based on the data available on the rest of the patients and the data on himself until the i^th^ inflammatory mediator reading. From these available data, we predict either a Low (poor outcome) or High (favorable outcome) value for GOS; the binary prediction can be refined as a function of the amount of data available. Since this method of analysis emphasizes patient profiling, the prediction of GOS takes this into evidence in the following way. After the i^th^ round of inflammatory mediator readings, each subject has an associated survival curve, as the ones highlighted in **Figure 2** for patients 11 and 14. The incoming patient has (up to the i^th^ inflammatory mediator reading) such a survival curve as well. We now select, from the data available on all other subjects (but not the incoming subject), the patient whose curve matches that of the incoming patient most closely. Closeness between two curves is measured by the difference of the area under them (up to and including the i^th^ inflammatory mediator reading). We predict the GOS for the incoming patient after the i^th^ inflammatory mediator reading to be the GOS (rounded to Low or High) of the patient(s) in the data base whose survival curve is closest to the curve of the incoming patient. The last inflammatory mediator reading on the incoming patient, based on the curve matching described above, yields the predicted GOS. For these data, we have a 72% success rate in prediction, a rate considerably higher than that of 50% obtained by assigning the GOS outcome to Low or High randomly, and one that is commensurate with other data-driven models for outcome prediction in TBI (Iverson et al., 2005).

## Discussion

Traumatic brain injury is a leading cause of morbidity and mortality in both the civilian and military settings. Like many other forms of injury, TBI is associated with an acute inflammatory response that drives, and in turn is likely driven by, further damage/dysfunction. The complexity of inflammation is daunting, and to date there have been no effective therapies that modulate inflammation in TBI (Namas et al., 2009).

After any injury, inflammation occurs as a necessary response, serving to remove or reduce challenges to the organism and subsequently restore homeostasis to promote organism survival. In an attempt to re-establish homeostasis, the inflammatory response clears foreign invaders and injured cells, enhances healing and promotes tissue repair. If sustained, the inflammatory response can also become excessive creating an environment that promotes further cell death (Medzhitov, 2008; Vodovotz et al., 2008). Tissue survival depends upon proper initiation and cessation of inflammation, mediated by pro- and anti-inflammatory mediator release. In response to trauma, pro-inflammatory mediators such as damage-associated molecular pattern (DAMP) molecules, chemokines, and cytokines are released to signal danger to injured cells (Namas et al., 2015). In the specific setting of head injury, TBI results in acute activation of astrocytes and microglia, with the release of pro- inflammatory mediators such as Interleukin (IL)-1β, IL-6, and Tumor Necrosis Factor (TNF)-α (Namas et al., 2009).

We and others have suggested that computational modeling is a means by which to integrate the numerous putative pathways known to be involved in post-injury inflammation and subsequent tissue damage/dysfunction. We have developed both mechanistic and data-driven computational models of inflammation in cells, experimental animals, and humans. We have suggested that such systems biology models could be used to simulate clinical trials, to predict the inflammatory responses of individuals, and to design novel drugs or devices for the control of inflammation (Vodovotz and An, 2013; Vodovotz and Billiar, 2013; Aerts et al., 2014).

In the present study, we utilized data-driven modeling to gain insights into the dynamic interactions among patient demographics, TBI severity and inflammation. We developed an algorithm, Dynamic Profiling, in order to integrate factors that clinicians would use in their decision making process (e.g., demographics, injury severity) along with biological data that, although currently not used in forming a diagnosis or prognosis, are thought to play a role in the pathophysiology of TBI. We envisioned this method as allowing for the estimation of probabilities of recovery for an individual patient at any time during the hospital stay based on the knowledge gained from the previously modeled data at all prior sampling times, knowledge gained by clustering subjects into disjoint groups and seeking predictive techniques that exploit both the similarities within clusters as well as differences among clusters. In this sense, Dynamic Profiling represents a form of precision medicine (National Research Council (U.S.). Committee on a Framework for Developing a New Taxonomy of Disease, 2011), in which data define the dynamic patient state. In another sense, Dynamic Profiling is a “virtual clinician”: the allocation of a given patient to a given cluster, and hence the likelihood to improve or decline in health, is carried out much as a clinician would. The initial assessment is based on patient demographics and injury characteristics. This diagnosis is refined over time as new data are obtained. In this particular instance, the data stream consists of CSF inflammatory mediators, since inflammation is considered to be a major driver of outcomes post-TBI, and since the CSF is the most proximal biofluid for measurement. Of course, data other than inflammatory mediators might be included in the dynamic profiling algorithm. For example, we have shown recently that we can incorporate biochemical and physiological data into data-driven network models of acute inflammation (Emr et al., 2014; Sadowsky et al., 2015).

We found that Dynamic Profiling could reach a predictive value of 72% with regard to GOS score. If validated further, we suggest that Dynamic Profiling could eventually be used in the treatment protocol for TBI patients. If the prediction from Dynamic Profiling is that of a high probability of non-survival, clinical intervention might be indicated. A new TBI patient may thus be steered, through clinical or surgical interventions, toward a cluster with as favorable a weight as possible. For example, **Figure 2** indicates that, perhaps at approximately the 6th or 7th round of inflammatory mediator readings, intense clinical intervention should have occurred to improve the probability of survival of Patient 14. Whether this would have been clinically viable, or even possible, is at present unknown, and therefore prospective studies are needed in order to validate this hypothetical approach.

Our study is subject to several limitations. First, the number of patients and the number of time points could be increased. Second, patient samples were constrained in part by the necessities of clinical management, and thus for some patients samples were missing. Another important limitation is that treatments were administered to the patients during the course of study but not registered in our data set; to do otherwise would have been unethical (or a more complete data collection could be performed for a future prospective study. Third, the panel of inflammation biomarkers assessed in each patient sample could be enlarged to encompass a broader set of mediators. Nonetheless, our findings point to a methodology that might be applicable in other complex, dynamic diseases.

## Author Contributions

GC, MB, QM, and FC Developed the dynamic profiling methods, ran logistical regressions, and calculated mathematical and statistical analyses. AA, RZ, AP, DO, and YV Coordinated clinical data collection, interpretation, and analyses and evaluated the results in the context of the clinical data.

## Conflict of Interest Statement

The authors declare that the research was conducted in the absence of any commercial or financial relationships that could be construed as a potential conflict of interest.

## Abbreviations

CBF: cerebral blood flow
CSF: cerebrospinal fluid
GCS: Glasgow Coma Scale
GOS: Glasgow Outcome Scale
IL: interleukin
MIP: macrophage inflammatory protein
TBI: traumatic brain injury
TNF-α: tumor necrosis factor-α
VEGF: vascular endothelial growth factor
